# Sequence‐discrete species for prokaryotes and other microbes: A historical perspective and pending issues

**DOI:** 10.1002/mlf2.12088

**Published:** 2023-12-11

**Authors:** Konstantinos T. Konstantinidis

**Affiliations:** ^1^ School of Civil and Environmental Engineering, and School of Biological Sciences Georgia Institute of Technology Atlanta Georgia USA

**Keywords:** average nucleotide identity (ANI), genomovar, homologous recombination, metagenomics, strain

## Abstract

Whether prokaryotes, and other microorganisms, form distinct clusters that can be recognized as species remains an issue of paramount theoretical as well as practical consequence in identifying, regulating, and communicating about these organisms. In the past decade, comparisons of thousands of genomes of isolates and hundreds of metagenomes have shown that prokaryotic diversity may be predominantly organized in such sequence‐discrete clusters, albeit organisms of intermediate relatedness between the identified clusters are also frequently found. Accumulating evidence suggests, however, that the latter “intermediate” organisms show enough ecological and/or functional distinctiveness to be considered different species. Notably, the area of discontinuity between clusters often—but not always—appears to be around 85%–95% genome‐average nucleotide identity, consistently among different taxa. More recent studies have revealed remarkably similar diversity patterns for viruses and microbial eukaryotes as well. This high consistency across taxa implies a specific mechanistic process that underlies the maintenance of the clusters. The underlying mechanism may be a substantial reduction in the efficiency of homologous recombination, which mediates (successful) horizontal gene transfer, around 95% nucleotide identity. Deviations from the 95% threshold (e.g., species showing lower intraspecies diversity) may be caused by ecological differentiation that imposes barriers to otherwise frequent gene transfer. While this hypothesis that clusters are driven by ecological differentiation coupled to recombination frequency (i.e., higher recombination within vs. between groups) is appealing, the supporting evidence remains anecdotal. The data needed to rigorously test the hypothesis toward advancing the species concept are also outlined.

## INTRODUCTION: MICROBIAL SPECIES MAY EXIST

Early studies in the 1980s and the 1990s based on DNA–DNA hybridization (DDH), the gold standard for species demarcation for prokaryotes^1^, ^2^, showed that organisms—mostly of the *Enterobacteriaceae* family—with >70% DDH to each other tended to have distinct phenotypic properties compared to their close relatives to which they typically show <70% DDH. This distinctiveness was viewed as adequate evidence to describe the former organisms as members of same (distinct) species and the 70% DDH standard has been the most influential threshold since then for (new) species descriptions. Our own subsequent work showed that the DDH values between two genomes most strongly correspond to the average nucleotide identity of their shared genes, which we termed as ANI, among several additional parameters derived from genome sequences that were evaluated^3^, ^4^. This discovery provided a convenient means to replace the laborious DDH technique with genome sequencing. Subsequently, ANI and similar genome‐derived metrics based on the ANI concept^5^, ^6^ have become popular and practically replaced DDH. Specifically, the 70% DDH standard corresponds strongly to a 95% ANI^3^.

Genome sequencing during the next two decades (i.e., 2000–2020), however, put the earlier findings based on DDH into question, in that genomes showing intermediate genomic relatedness values, for example, 90%–95% ANI corresponding to 30%–70% DDH, and overlapping phenotypes compared to representative genomes of known/named species were commonly found^7^, ^8^. This is best exemplified by the *Escherichia coli* group, the model bacterial species, where several genomes that share the diagnostic phenotype of *E. coli* (e.g., fermentation of lactose) became available around 2010 and show ANI values to *E. coli* type genomes in the 90%–92% range^9^. These and similar findings led several scientists to suggest that bacterial species may not exist^10^, ^11^ and/or that the boundaries between species are “fuzzy” at best^8^. Another important finding from the first genome sequences that became available at the beginning of the century was that bacterial genomes are much more fluid than previously thought and often engage in extensive horizontal (contrasting with vertical) gene transfer (HGT). These early studies have also shown that not all genes in the genome undergo HGT at high frequency. Specifically, universal genes, that is, those shared by almost all microbial genomes and encoding such proteins as the ribosomal proteins and DNA/RNA polymerases, are infrequently transferred horizontally primarily because their transfer does not usually provide a selective advantage to the recipient organism relative to the native homolog genes present in the genome^12^, ^13^. Hence, it is possible to build a backbone genealogy (or species tree) based on the latter genes, which mostly encode universal functions shared by all microbes (for a contrasting view, see Doolittle^10^). The high frequency of HGT observed in the bacterial domain, however, led several scientists to reinforce the idea of species‐less bacterial diversity^10^, ^14^. Furthermore, inspired by the infrequent horizontal transfer of universal marker genes, several methods based on the phylogenetic analysis of these markers have been developed to catalog prokaryotic species diversity such as the GTDB^7^, PhyloPhlAn^15^, and mOTU^16^ webservers. These methods are largely consistent with the ANI approach on the intragenus level^17^, and thus can be used interchangeably with ANI, although the ANI approach may provide higher resolution among closely related genomes (e.g., of the same or closely related species) in some cases due to the higher sequence conservation of the universal genes relative to the genome average (represented by ANI)^18^. Hence, the ANI results for assessing the species level are preferably reported below (also note that ANI is a metric that is robust to the horizontal transfer of a few, or even tens, of genes, and easier to appreciate compared to distances based on maximum likelihood or other algorithms).

Contrasting with the genome‐based findings reported in the 2000–2010 era of highly dynamic (or plastic) bacterial genomes and fuzzy species, more recent metagenomic surveys of natural populations based on shotgun sequencing of environmental DNA^19^, ^20^ revealed that these populations are sequence‐discrete and that the level of discreteness is typically observed around 95% ANI^21^. That is, members of the same, sequence‐discrete population typically share >95% ANI among themselves and <90% ANI with any co‐occurring organisms in the sample. While this pattern was first observed by us in the deep ocean^21^, one of the most stable environments on the planet, more recent work by us and others showed that similar diversity patterns exist in other environments^22^, ^23^, ^24^. Further, by sampling the same environment over time (time‐series) or the same environment (ecological niche) across distant locations, we have shown that the sequence‐discrete populations are not ephemeral but long‐lived entities, and thus have the properties expected of species^25^, ^26^. Genomes of intermediate identity were often—but not always—present in these environmental surveys, but they appeared to be ecologically differentiated based on the fact that, in the same sample, they were typically much less abundant than the dominant population assessed by metagenomics. The differential abundance under the same growth conditions (same sample) coupled to substantial genomic divergence, for example, at least 5%–15% difference in ANI values, which is typically accompanied by 20% or more different gene content, and thus presumably substantial phenotypic differences^27^, argued that such intermediate genomes should be considered members of different species or, at least, on their way to speciation. Finally, it should be mentioned that while sequence divergence and ecological and/or functional differentiation appear to go together in the cases mentioned above, this should not be expected to be the case always. For instance, the recent acquisition of a functional module that allows a subpopulation to explore a new niche within an otherwise identical genomic background relative to the main population could represent the initial stage of speciation and a case where genome relatedness and functional differentiation may depart from one another. However, given enough evolutionary time, this ecological differentiation would also start becoming obvious at the sequence relatedness level. Therefore, distinct “species” might be found within the 95% ANI clusters, although these are much more challenging to detect unequivocally based on genome/metagenome comparisons and distinguish from intraspecies units such as genomovars and strains (further discussed below). For the latter reasons, it may be more pragmatic, in general, not to describe groups of genomes that have not differentiated enough yet at the sequence level (of shared genes) as new species and instead refer to such groups as distinct intraspecies units.

**Figure 1:**
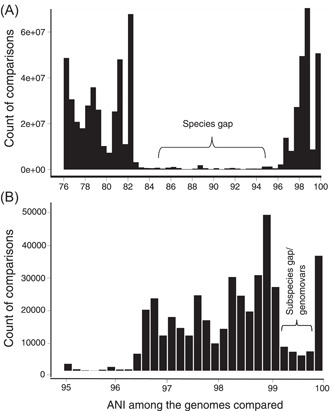
The average nucleotide identity (ANI) gaps at the species and subspecies levels. The histograms are based on pairwise comparisons performed with FastANI between about 90,000 complete genomes available in the NCBI database as described previously^18^. The graphs show the number of genome pairs among the total pairs compared (*y*‐axes) plotted against their whole‐genome ANI values (*x*‐axes). The graph in (A) (top; species level) was drawn based on the data available in Jain et al.^18^, while the graph in (B) (bottom; subspecies level) was drawn based on the data in Rodrigues‐R et al.^28^. Note the clear gap (of discontinuity) between species in the 84%–96% ANI range (i.e., shortage of genome pairs showing values in this range relative to genome pairs showing >96% or <84% ANI) as well as the less pronounced but still clearly noticeable gap within species in the 99.2%–99.8% ANI range (mean 99.5%).

**Figure 2:**
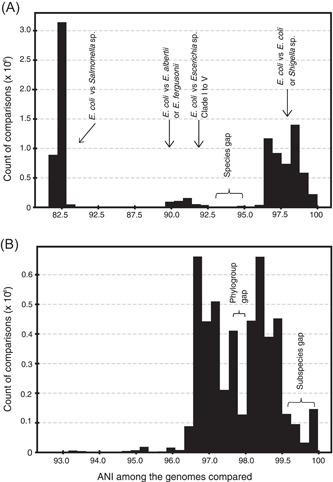
The average nucleotide identity (ANI) gaps at the species and subspecies levels for the *Escherichia coli* group. This figure is identical to Figure 1, except that it shows only data from the model bacterial species, *E. coli*. The graph in (A) (interspecies) shows data from comparisons of all *E. coli* genomes against themselves or genomes of their close relatives, while the graph in (B) (intraspecies) includes only comparisons among genomes assigned to *E. coli*. The number of genomes used is as follows: *E. coli*, 2815; *Salmonella enterica*, 1351; *Escherichia fergusonii*, 57; *Escherichia albertii*, 70; and *Shigella flexneri*, 93 (all complete genomes available at NCBI as of July 2023). Additionally, representative draft genomes of the environmental *Escherichia* clades I to V reported previously^9^ were included in (A) (denoted on the graph). Note that both the species gap around 95% ANI and the subspecies at around 99.5% are obvious, albeit the latter may not be as pronounced as that observed in Figure 1B based on all highly sampled species because not many closely related *E. coli* genomes have been completed. However, note also that the 99.5% ANI gap is as pronounced, when compared to its adjacent bars, as is the 98%–97% ANI gap that corresponds to the gap between *E. coli* phylogroups, a well‐recognized, distinct grouping within *E. coli* ^29^.

More recent analysis of all available complete genomes in NCBI database (*n* = 90,000) by our team has shown—consistent with the 70% DDH definition of species—that the great majority of described (named) species (~97% of the total) include genomes showing >95% ANI among themselves versus <86% ANI to representatives of other species (Jain et al.^18^ and Figure 1; Figure 2 shows the data from the *E. coli* group only as an example). That is, the picture emerging from these isolate genome comparisons is strikingly similar to the findings from metagenomics and different from the earlier findings based on isolate genome sequencing mentioned above. The qualitative difference from the earlier isolate‐based findings may be due to the fact that organisms with adaptations to different ecological niches were compared previously, and thus revealed indiscrete species. For instance, in the *E. coli* example mentioned above, genomes showing 90%–92% ANI to *E. coli* type genomes are rarely recovered from the human gut (unlike typical *E. coli*), and instead are thought to occupy the gut of birds and other animals or even environments outside a host^9^. When organisms that occupy the same ecological niche are compared as assessed via metagenomic sequencing, or the analysis is focused on the great majority of isolate genomes available in the database (as opposed to a few selected genomes), sequence‐discrete species become obvious^27^. Perhaps more importantly, the area of discreteness between sequence‐discrete clusters matches almost perfectly how organisms have been assigned to species historically, even before the invention of genome sequencing, suggesting that the existing system (taxonomy) is robust and predictive of organism relatedness, at least at the species level^18^.

A few concerns have been raised recently that the genetic discontinuity between sequence‐discrete species may be due to an artifact of undersampling of genomes of intermediate identities due to isolation or other biases^7^, ^30^. However, the metagenomic surveys are believed to be free of such biases and the findings from such surveys strongly support the low frequency of intermediate genotypes. Further, more recent large‐scale isolation efforts from the same environment, such as of *Salinibacter ruber* from hypersaline sites across the globe, have consistently recovered a low frequency of, if any at all, intermediate genotypes^31^, ^32^. In summary, it seems that bacterial species may exist, and the 95% ANI threshold represents a general‐purpose and pragmatic threshold to describe them. It should be mentioned, however, that younger species since the last diversity sweep may include more genomically homogeneous members, and so the ANI threshold (or area of discreteness) for them appears to be higher (e.g., around 98% ANI). Further, a few species have also been observed for which the 95% ANI may be too stringent. Most notable are the most abundant photosynthetic organism, *Prochlorococcus marinus*, and the most abundant heterotroph, *Pelagibacter ubique*, in the oceans, which harbor members that often show around 92%–93% ANI^21^, ^33^. Therefore, for future species descriptions, or re‐evaluation of existing (named) species, it is recommended to assess the ANI value distribution among members of the proposed species and their close relatives and adjust the ANI threshold to match the areas of discreteness if the diversity patterns indicate to do so^27^, ^34^. It is remarkable that the most commonly observed area of discreteness, around 95% ANI, corresponds strongly to the traditional 70% DDH (described above). The high consistency between the recent genomic‐ and metagenomic‐based findings with those from the “pregenomics” era implies that there is a fundamental mechanism that underlies the sequence‐discrete species, the effect of which (i.e., genomic and phenotypic distinctiveness among species) has been observed for at least five decades now. The probable mechanisms are examined in the next section.

It is also important to note that similar sequence‐discrete species to those described above for *Bacteria* have recently been recognized for *Archaea* and other microbes, most notably, eukaryotic protozoa^35^, and different types of viruses, including bacteriophages, and DNA‐ or RNA‐based viruses of eukaryotic hosts^36^, ^37^. For example, our own work with eukaryotic protozoa has revealed several cryptic species based on genomic discreteness using ANI or other genome‐based methods within the named *Giardia duodenalis*, which was previously defined based on traditional methods characterized by coarse resolution^35^. Viral species are commonly defined based on the 95% ANI threshold nowadays because the latter has been found to correspond to the area of discreteness between previously named species or the clusters of diversity revealed by metagenomic approaches^36^. Therefore, it appears that similar species‐level diversity patterns may characterize most microbes, including viruses.

## WHAT ARE THE MECHANISMS OF COHESION FOR THE SEQUENCE‐DISCRETE SPECIES?

The high consistency in terms of the 90%–95% ANI representing the area of discreteness between closely related species across different microbial taxa implies that a common mechanism(s) may underlie these diversity patterns. While random death and birth (speciation) could provide sequence‐discrete clusters similar to those described above (Figure 1)^38^, obtaining clusters with the area of intercluster discreteness to be centered around the exact same ANI value (i.e., 90%–95%) across taxa with different lifestyles (e.g., symbiotic vs. free‐living), habitats, or even informational material (e.g., DNA vs. RNA viruses) just based on random processes seems rather unlikely. Instead, a genetic (e.g., recombination, selection for specific mutations) and/or an ecological mechanism (e.g., functional differentiation in response to changing growth conditions) must be at play. Among the possible mechanisms, recombination frequency (e.g., being higher within vs. between clusters) has gained popularity among several scientists^39^, ^40^, ^41^, ^42^, but it can be argued that the data in support of this mechanism reported in the literature thus far are anecdotal.

**Figure 3:**
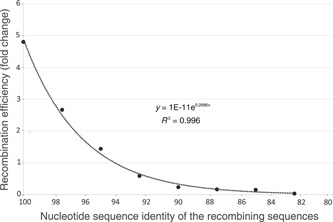
Efficiency of homologous recombination as a function of the nucleotide identity of the recombining sequences. The figure is drawn based on the whole‐genome transformation data available in Power et al.^45^, and shows the efficiency of recombination, measured as the likelihood of two sequences to recombine (*y*‐axis), plotted against the nucleotide sequence identity of the two sequences (*x*‐axis). For instance, for sequences that are 100% identical, there is about fivefold higher chance (or likelihood) for such sequences to recombine compared to sequences that show about 94% identity. The chance was estimated as follows based on the data reported in fig. 1of Power et al.^45^: the total number of sequences in the recipient genome showing 100% identity to the donor DNA before the transformation experiments made up about 5% of the total genome but, after transformation, these sequences made up about 24% of the total sequences that were found to recombine, providing a 4.8‐fold higher frequency for recombination than expected by chance alone based on the prevalence of such sequences in the genome before transformation (i.e., 24/5 = 4.8). The same value for the sequences showing 94% identity was about 1, providing a fivefold higher chance for recombination for the former relative to the latter sequences. Note that the efficiency of recombination is more than one order of magnitude lower for sequences showing about 90% identity compared to sequences showing 100% identity.

Bacterial genomes engage in frequent HGT as mentioned above and as documented in many publications. For example, genomes of the same (named) species frequently differ in 20%–30% of their genes and most of these gene‐content differences are driven by HGT and gene deletion^43^. A transferred gene or piece of DNA needs to be integrated into the genome of the recipient cell in order for the HGT event to be successful (reviewed in Gogarten and Townsend^12^). DNA integration could occur via a homologous recombination mechanism, where the foreign DNA replaces a highly identical DNA region in the recipient genome (90% nucleotide identity or more is usually required; Figure 3) mediated by the *RecA* protein complex. When the foreign DNA is not similar enough to the recipient genome, integration can occur via a nonhomologous mechanism, mediated by a different enzyme complex that cuts the recipient genome and integrates the foreign DNA into it (e.g., an integrase or transposase system). For species cohesion, homologous recombination is more important because the nonhomologous mechanism typically yields new gene functions as part of the dissimilar foreign DNA that is integrated into the recipient genome, and thus could lead to functional diversification and diversity loss (speciation) rather than species cohesion. What has made HGT mediated by homologous recombination (or just “homologous recombination” for simplicity) an attractive hypothesis to explain sequence‐discrete clusters is earlier laboratory studies in which specific (single‐gene) point mutations were complemented via the transformation (electroporation) of a recipient cell with foreign DNA of known sequence identity (e.g., Vulic et al.^44^). These studies showed that the frequency of recombination decreases substantially around 95% nucleotide identity, albeit recombination is still possible even among pieces of DNA that are less than 90% identical, depending on the secondary structure of the recombining DNA, among other factors. More recently, high‐throughput transformation studies at the whole‐genome level have further corroborated these earlier findings and showed that homologous recombination efficiency decreases by fivefold or more for sequences that are 94%–95% nucleotide identical relative to those that have 99%–100% identical^45^. This study has also shown that the homologous recombination efficiency decreases to almost zero around 90% identity (Figure 3), which intriguingly corresponds to the highest intraspecies ANI diversity level observed (92%–100% ANI) for the long‐lived marine taxa mentioned above. While such findings have indicated that homologous recombination could be a possible mechanism underlying the diversity patterns observed at the species level, there are two additional requirements that need to be satisfied in order for homologous recombination to represent the force of species cohesion. (i) Homologous recombination has to remove more sequence diversity than point mutations and other types of diversifying mutations (e.g., indel insertions) brought into the genome within the same evolutionary time, that is, to be *frequent enough*. (ii) There should not be any (sizeable) regions of the genome where the recombination frequency is too low or even absent for any reason because such areas will continue to diverge due to point mutation accumulation, that is, to be *random (unbiased) enough* across the genome.

The effect of recombination on genome evolution has been assessed based primarily on two sets of methods (or approaches) or hybrid methods based on these two approaches (recently reviewed in Shikov et al.^46^). The first set is based on reconstructing ancestral sequences using maximum likelihood or Bayesian approaches and some expectation of the effects of recombination versus point mutation on sequence identity and testing these reconstructions against the phylogenetic tree of the actual (available) gene sequence of interest (e.g., Didelot and Falush^47^ and Kosakavsky Pond et al.^48^). The advantage of this approach is that it can include historic recombination (older events), in addition to recent events, and it is based on a theoretical framework. However, the results of this approach have been met with skepticism, in general, because its assumptions likely violate some principles in gene/genome evolution, most notably the effects of (positive) selection. For example, let us assume that the same gene is compared between three organisms, A, B, and C, and was found to have a single point mutation (single nucleotide substitution or SNP) in 1000 otherwise identical nucleotide positions (the average length of bacterial genes) that is shared by A and B but not C. Recombination detection software would most likely call this a recombination event between A and B because, mathematically, it is highly unlikely for two sequences to share the exact same SNP within a background of 1000 nucleotide positions. That is, if this substitution was caused by a point mutation instead, the mutation could have occurred in any of the 1000 available positions, and thus would have been highly unlikely to be shared by A and B (1 in 1000) (there are also three possibilities for different nucleotide substitutions that would result in an SNP at that position). However, strong selection for a specific substitution due to a gain of function or another selective advantage could also account for the (hypothetical) scenario described above as illustrated repeatedly by experimental evolution of independent and nonrecombining *E. coli* lineages for >60,000 generations under laboratory settings^49^. Furthermore, systematic sequencing errors could also account for similar patterns in nucleotide substitutions. It is particularly challenging to account for the effects of selection on sequence evolution especially under variable environmental conditions, which could cause the selection pressure for specific mutations to change over time. Such variable environmental conditions as well as selection interference between independent mutations in different parts of the genome may be quite common in most environments and can confound recombination detection efforts.

To circumvent this challenge, a second set of methods examines sequence identity patterns and aims to detect highly identical sequences over a background of more variable genome sequences (e.g., Bobay and Ochman^40^ and Arevalo et al.^50^). Therefore, this approach has fewer underlying assumptions and focuses on more recent recombination events but cannot assess historic recombination. The latter is, at least partly, due to the fact that recombined segments are usually hotspots for accumulation of subsequent mutations in order to ameliorate the foreign sequence to the codon biases of the recipient genome (when codon biases of the donor genome differ, which is often the case). The larger the difference of the signal (e.g., 100% identical sequences for recombining segments) over the noise (e.g., background genome showing 95% ANI or less), the stronger the evidence in support of recent recombination. Application of this approach to large collections of genomes and metagenome‐assembled genomes (MAGs) has confirmed earlier observations that bacterial genomes are fluid and often undergo extensive HGT and recombination^40^, ^41^. Consequently, these studies have concluded that the frequency of homologous recombination could be high enough to have a greater effect on sequence evolution than point mutation at the whole‐genome level, as has been shown to be the case for *Campylobacter* species^42^ and other taxa more recently^40^. However, these studies have not been able to assess whether recombination is random enough across the genome to serve as a force of cohesion. In fact, at least in the *Campylobacter* case, recombination was convincingly shown to be biased to a few specific regions (genomic islands) of the genome and functions (e.g., antibiotic resistance and motility) that are apparently under positive selection. Thus, recombination is unlikely to lead to species cohesion in such cases or convergence of closely related *Campylobacter* species^51^. Another challenge with this type of analysis is that, at this intraspecies level, the signal (e.g., 100% identity recombined genes) may sometimes be too close to the background (e.g., ANI among some genomes of a species could be 99% or higher) to make confident calls of recombination detection, especially for highly clonal species that do not harbor extensive sequence diversity. Moreover, at least a few genes in the genome such as the ribosomal RNA operon genes and several ribosomal protein genes are highly conserved at the sequence level due to strong functional constraints on their nucleotide sequence (or 3‐D structure for protein‐coding genes). Therefore, the high sequence identity of these genes between closely related genomes may be due to these constraints rather than recent recombination^52^. Hence, the question of whether or not recombination is frequent and random enough across the genome to maintain the sequence‐discrete clusters remains to be more rigorously tested in the future.

One additional complication arises from the effect of ecology on recombination frequency. The lack of recently recombined genes between any two genomes has been taken as evidence that the genomes are sexually isolated and thus should represent different species^40^. However, lack of recent gene exchange could reflect ecological differentiation rather than the direct effect of genetic isolation or incompatibility (caused—for instance—by different DNA methylation patterns or increased sequence divergence)^53^. For instance, if two populations become isolated in their respective environments and there is no effective dispersion between the environments, they would cease to recombine and would speciate allopatrically. While most microbial cells can be found everywhere, as the recent SARS‐CoV‐2 pandemic has also confirmed, allopatric speciation has been shown to occur for at least several obligatory microbes of eukaryotic hosts and organisms of hyperthermal vents that show limited dispersion^54^. Further, if the cells are dormant in a given environment, they are unlikely to engage in genetic exchange. Hence, differential growth within the same environment could also lead to (ecological) speciation sympatrically, although such cases are more challenging to detect and confirm in general (e.g., see Strachan et al.^55^). It is also important to realize that the 5% nucleotide difference in shared genes (corresponding to 95% ANI) represents thousands of generations (and thus years) since the last common ancestor^56^. Within such long timeframes, ecological speciation driven by dispersion limitation or by gain of a new function could lead to exploration of a new ecological niche. These scenarios would lead to ecological, as opposed to sexual/genetic, speciation and it is important to separate the two cases for advancing the species concept because the underlying mechanism is fundamentally different, even if the ultimate outcome (e.g., emergence of species‐level clusters) could be similar.

## AN ANI GAP WITHIN SPECIES TO DEFINE SUBSPECIES UNITS

Our more recent analysis of 300 bacterial species with more than 10 sequenced representatives each has revealed a second ANI discontinuity (or gap) within species and consistent across species, similar to the 95% ANI gap at the species level, which can be used to provide reliable subspecies resolution (Figure 1 and Rodrigues‐R et al.^28^). Notably, discrete, or somewhat discrete^8^, ecological or evolutionary units within bacterial species have long been recognized and are designated by various terms such as ecotypes, clonal complexes, sequence types, and serotypes, among several other terms (recently reviewed in Rossello‐Mora and Amann^57^). However, the application of these units has been largely inconsistent between different species, for example, different marker genes and standards for each marker are used, making the subspecies units not easily comparable between different species. The 99.5% ANI gap appears to closely match the definition of sequence types (STs), for example, ~80% of the genomes assigned to the same ST also share >99.5% ANI, on average. STs are collections of genomes sharing no sequence divergence in six to seven loci (typically intragene regions) and have been used commonly in medical microbiology and epidemiology to track outbreaks caused by specific organisms^58^. Our initial analysis of several highly sampled bacterial pathogens has shown that the 99.5% of ANI‐based clusters are substantially more homogeneous in terms of the gene content and sequence diversity of the grouped genomes compared to their corresponding STs^28^. Hence, the 99.5% ANI level could be used to define STs more reliably and consistently across taxa and/or refine existing STs. However, because STs have their own historic value in communication, it is proposed to refer to the 99.5% ANI clusters as genomovars instead. The term genomovar was originally used to name distinct genomic groups within species that cannot be distinguished phenotypically from each other in order to be named as distinct species^59^. Hence, genomovar may best capture the 99.5% ANI units conceptually.

No other pronounced ANI gap within the 300 evaluated species was consistently observed, and thus we recommend the use of the term strain only for nearly identical genomes. Specifically, we propose to define a strain as a collection of genomes sharing ANI > 99.99% based on the high gene content similarity observed at this level based on the genomes compared; for example, typically, >99.0% gene content is shared (Viver et al.^31^ and Table 1). It should be mentioned, however, that a 1% gene content difference for a 5 Mbp genome (e.g., *E. coli*'s average genome size) translates to 50 genes, which represents a substantial (nontrivial) gene content difference and could underlie significant phenotypic differences such as different antibiotic resistance profiles and plasmid DNA content^28^. We suggest letting the ANI > 99.99% threshold override gene‐content differences in order to simplify strain identification and communication. The gene content differences at this (strain) level are also typically carried by mobile elements (e.g., plasmids, transposons), and thus do not represent stable properties of the genome. In summary, we suggest evaluating the intraspecies ANI value patterns and using them to define genomovar and strain levels toward more data‐informed subspecies units than the current practice. The 99.5% and 99.99% ANI thresholds for genomovars and strains, respectively, should work in most cases based on the available data analyzed thus far. However, we also recommend adjusting these values as necessary in order to better match the ANI value gap observed for the species of interest and/or highly important phenotypic differences identified for strain designations; for example, in a few species, the 99.5% ANI may be shifted downward around 99.0% or 99.2% ANI^28^.

**Table 1:** Recommended average nucleotide identity (ANI) and amino‐acid identity (AAI) thresholds for key taxonomic ranks.

Taxonomic rank	ANI/AAI threshold
(Same) Strain	ANI > 99.99%, gene‐content >99% (typical)
Genomovar or sequence type	ANI > 99.5%, gene‐content > 90% (typical)
Species	ANI > 95.0%, gene‐content > 70% (typical)
Genus	AAI > 65.0%, gene‐content > 50% (typical)
Family	AAI > 45.0%, gene‐content > 20% (typical)

For pairs of genomes showing <80% ANI and/or having <10% of the total genes or fragments in the genome shared, it is recommended to switch to the AAI of shared genes because ANI values are typically not reliable at this level. Also note that the proposed thresholds are largely consistent with those proposed previously for the same ranks (e.g., species or genus)^60^, ^61^, ^62^, and slight differences in the thresholds may be due to the exact tools used to calculate ANI. For instance, ANI based on universal genes is typically higher than ANI based on the whole genome^18^.

## LOOKING FORWARD: WHAT IS STILL NEEDED?

The exact mechanisms underlying the 99.5% ANI gap, or the previously established 95% ANI for the species level, remain speculative, although they are likely related somewhat to ecological differentiation, coupled to recombination frequency; for example, higher HGT mediated by homologous recombination within versus between clusters. These mechanisms should be the subject of future research toward advancing the species concept for bacteria and other microbes. It is also possible that different mechanisms may underlie the speciation of different taxa due to varied contributions of functional gene content differentiation, dispersion, frequency of recombination, genetic compatibility, or other processes. To quantitatively test the role of these mechanisms, several closely related species should be followed over relevant environmental fluctuations using metagenomics and whole‐genome sequencing based on high‐throughput isolation and/or single‐cell genomics. By measuring the extent of homologous recombination among the isolate genomes of the same versus distinct but very closely related populations sharing 85%–95% ANI, as well as at the beginning versus the end of the sampling period within a species, the importance of recombination should be quantifiable. Such studies should be performed in the natural environment of the organisms in order to provide realistic measurements relative to those that can be obtained from artificial laboratory growth conditions. One challenge with this approach is that it might take too many generations (time) to see the effect of recombination and the exchanged genes to become fixed, or even detectable, in the population. Therefore, alternative approaches that are focused on bioinformatics analysis of genomes already available in the databases should also be pursued. While there are several recombination detection tools available, their application to available genomes should be performed with caution, especially with respect to the frequency of recent recombination events (which are most relevant for species cohesion) and the (lack of) spatial bias across the genome as described above. It is also important to distinguish between older (historical) recombination events and more recent events, as the latter may be relatively more important for assessing genomovar and species cohesion at the present time.
